# Development and implementation of the SUM breast cancer cell line functional genomics knowledge base

**DOI:** 10.1038/s41523-020-0173-z

**Published:** 2020-07-21

**Authors:** Stephen P. Ethier, Stephen T. Guest, Elizabeth Garrett-Mayer, Kent Armeson, Robert C. Wilson, Kathryn Duchinski, Daniel Couch, Joe W. Gray, Christiana Kappler

**Affiliations:** 1 Department of Pathology and Laboratory Medicine, Medical University of South Carolina, Charleston, SC USA; 2 Biostatistics Core, Hollings Cancer Center, Charleston, SC USA; 3 Department of Computer Science, The College of Charleston, Charleston, SC USA; 4 Department of Biomedical Engineering, Oregon Health and Sciences University, Portland, OR USA; 5 Present Address: Department of Biomedical Informatics, University of Michigan Medical School, Ann Arbor, MI USA; 6 Present Address: American Society for Clinical Oncology, Charleston, SC USA; 7 Present Address: Program in Bioinformatics and Integrative Genomics, Harvard University, Cambridge, MA USA

**Keywords:** Cell signalling, Cancer genomics

## Abstract

Several years ago, the SUM panel of human breast cancer cell lines was developed, and these cell lines have been distributed to hundreds of labs worldwide. Our lab and others have developed extensive omics data sets from these cells. More recently, we performed genome-scale shRNA essentiality screens on the entire SUM line panel, as well as on MCF10A cells, MCF-7 cells, and MCF-7LTED cells. These gene essentiality data sets allowed us to perform orthogonal analyses that functionalize the otherwise descriptive genomic data obtained from traditional genomics platforms. To make these omics data sets available to users of the SUM lines, and to allow users to mine these data sets, we developed the SUM Breast Cancer Cell Line Knowledge Base. This knowledge base provides information on the derivation of each cell line, provides protocols for the proper maintenance of the cells, and provides a series of data mining tools that allow rapid identification of the oncogene signatures for each line, the enrichment of KEGG pathways with screen hit and gene expression data, an analysis of protein and phospho-protein expression for the cell lines, as well as a gene search tool and a functional-druggable signature tool. Recently, we expanded our database to include genomic data for an additional 27 commonly used breast cancer cell lines. Thus, the SLKBase provides users with deep insights into the biology of human breast cancer cell lines that can be used to develop strategies for the reverse engineering of individual breast cancer cell lines.

## Introduction

Over the past decade, many genomic data sets have been obtained from breast cancer cell lines, primary xenograft models of breast cancer, primary breast cancer specimens, and most recently, from metastatic lesions. The types of genomic data that are now available to clinicians and laboratory scientists include sequencing-based analyses that show the point mutations that occur in breast cancer, copy number data that provide information on the oncogenes activated by gene amplification as well as the tumor suppressor genes inactivated by point mutations or homozygous deletion, gene expression data from RNA-sequencing and gene expression arrays, and most recently, proteomics and phospho-proteomics data. In addition, a number of tools and databases have been developed, such as the CaBio Portal^1^, the Kaplan–Meyer plotter^2,3^, the DepMap portal^4^, and others that allow breast cancer researchers to mine these data sets and draw inferences about the influence of specific genomic changes on breast cancer development, progression, and outcome.

As powerful as these tools and data sets are, they are descriptive in nature and the inferences and conclusions that can be drawn from them are, as a result, correlative. Recently, a number of laboratories, including our own, have performed shRNA or CRISPR-Cas9-based gene essentiality screens on a large number of human breast cancer cell lines. Some of these screens have been focused on specific gene sets, such as the kinome^5,6^, whereas others have been genome-scale and made use of libraries of varying complexities^4,7–9^. We have performed genome-scale shRNA screens for the SUM breast cancer cell line panel and some of the results from those experiments have been reported^10–12^. These functional screens have resulted in the generation of long lists of genes that have been found to play a direct role in either the proliferation or survival of specific human breast cancer cell lines. Moreover, when these sets of essential genes are analyzed with respect to the descriptive genomic data sets described above, the result is a functionalization of the genomic data that sheds light on the biology of individual breast cancer cell lines. The functionalization of genomic data is particularly important with respect to predicting the sensitivity or resistance of breast cancer cells to targeted drugs. Targeted drugs are effective when they inactivate a functional driver gene, but they are ineffective when they target a passenger gene, and this notion is supported by results of numerous clinical trials, both positive and negative. Because gene essentiality screens effectively distinguish driver from passenger genes, regardless of expression level or genomic status, these analyses are powerful ways to identify druggable targets in breast cancer cells. Thus, functional genomics has the potential to make accurate predictions about targeted drugs that are likely to have the largest impact on cancer cells with the highest therapeutic index.

To take full advantage of these functional genomics data sets, new tools need to be developed that allow breast cancer researchers to quickly identify the most essential genes, oncogenes, pathways, and druggable targets for any cell line, and then ultimately translate that knowledge to make predictions about breast cancer specimens from primary sites or metastatic lesions. Over the past 5 years, our laboratory has performed genome-scale shRNA screens on the SUM breast cancer cell lines developed in our laboratory, as well as on MCF10A cells, MCF-7 cells, and MCF-7LTED cells^10–12^. More recently, we developed a series of web-based tools that allow breast cancer researchers who work with these cell lines to quickly and easily mine the data to identify the functional oncogenes, the most essential biological pathways, and the genes within those pathways that mediate growth and survival of the cells, as well as the best druggable targets in each cell line based on essentiality and druggability of each gene. Other data mining tools allow researchers to determine the status of any annotated gene in the genome in any cell line, along with its expression status at the mRNA and protein level, and genomic alterations that may be present in the gene. Finally, all of these data and data mining tools are presented in the context of a Knowledge Base that provides critical information on the derivation of each cell line, the patient from which the cells were derived, and the proper conditions for maintaining each cell line. Recently, we have expanded this Knowledge Base to include 27 additional breast cancer cell lines for which reliable genomics and functional screening data are available, and the data mining tools originally developed for the SUM lines can be used for a total of 40 breast cancer cell lines.

This Breast Cancer Cell Line Knowledge Base and the data mining tools contained within allow for rapid functional genomic analysis not only for the SUM breast cancer cell lines but for all breast cancer cell lines for which validated functional screens have been performed.

## Results

### Rationale for development of the SLKBase

Human breast cancer cell lines have been and continue to be a mainstay of breast cancer research worldwide. Indeed, breast cancer cell lines have played a key role not only in helping to elucidate the fundamental biology of breast cancer but also for the development of virtually every drug that is used to treat breast cancer patients. The MCF-7 cell line was critically important to the development of the hormonal therapies used to treat patients with estrogen-receptor positive breast cancer^13–16^, as were breast cancer cell lines with *HER2* amplifications for the development of HER2-targeted drugs^17–24^. More recently, palbociclib was identified in a drug screen using a large panel of breast cancer cell lines^25^. Despite the importance of breast cancer cell lines in the development of modern therapeutic modalities for breast cancer, most researchers know relatively little about the cell lines they work with, and thus, the full potential of the large panel of breast cancer cell lines that currently exists has not been fully realized. In attempt to address this gap in our understanding, and to increase the power and importance of breast cancer cell lines in research, we set out to develop a knowledge base that allows researchers using the SUM breast cancer cell lines, as well as other commonly used breast cancer cell lines, to have ready access to the genomic and functional genomic data that have been generated from these cells, and to be able to quickly and easily mine these complex data sets. The SUM Breast Cancer Cell Line Knowledge base is the result of these efforts and provides a gateway for the functional genomic analysis of breast cancer cell lines.

### Development of a MySQL breast cancer genomics database

There were three overarching goals in the original development of the SLKBase: (1) to provide a rich source of information for anyone working with any of the SUM breast cancer cell lines, (2) to give researchers ready access to the large genomic data sets that have been developed with these cells, and (3) to allow researchers to perform orthogonal analyses of the various genomics data sets that we and others have obtained from the SUM lines. To build a platform for analysis of genomic data sets from the SUM lines, we first built a MySQL database that contains copy number data derived from array comparative genomic hybridization, gene expression data derived from Illumina bead arrays and more recently from RNA sequencing, point mutation data derived from whole-exome sequencing, and finally, data from the genome-scale shRNA screens for each of the SUM lines and for MCF10A, MCF-7, and MCF-7LTED cells^26^. In addition, we incorporated into the database the list of targeted drugs that are linked to specific genes from the Genomics of Drug Sensitivity in Cancer database. We then designed and launched a series of web-based tools that allow these data sets to be mined in ways that shed light on the deep biology of each cell line and suggest targeted drug strategies that are likely to be effective in each of the lines.

### Oncogene signatures

**Fig. 1:**
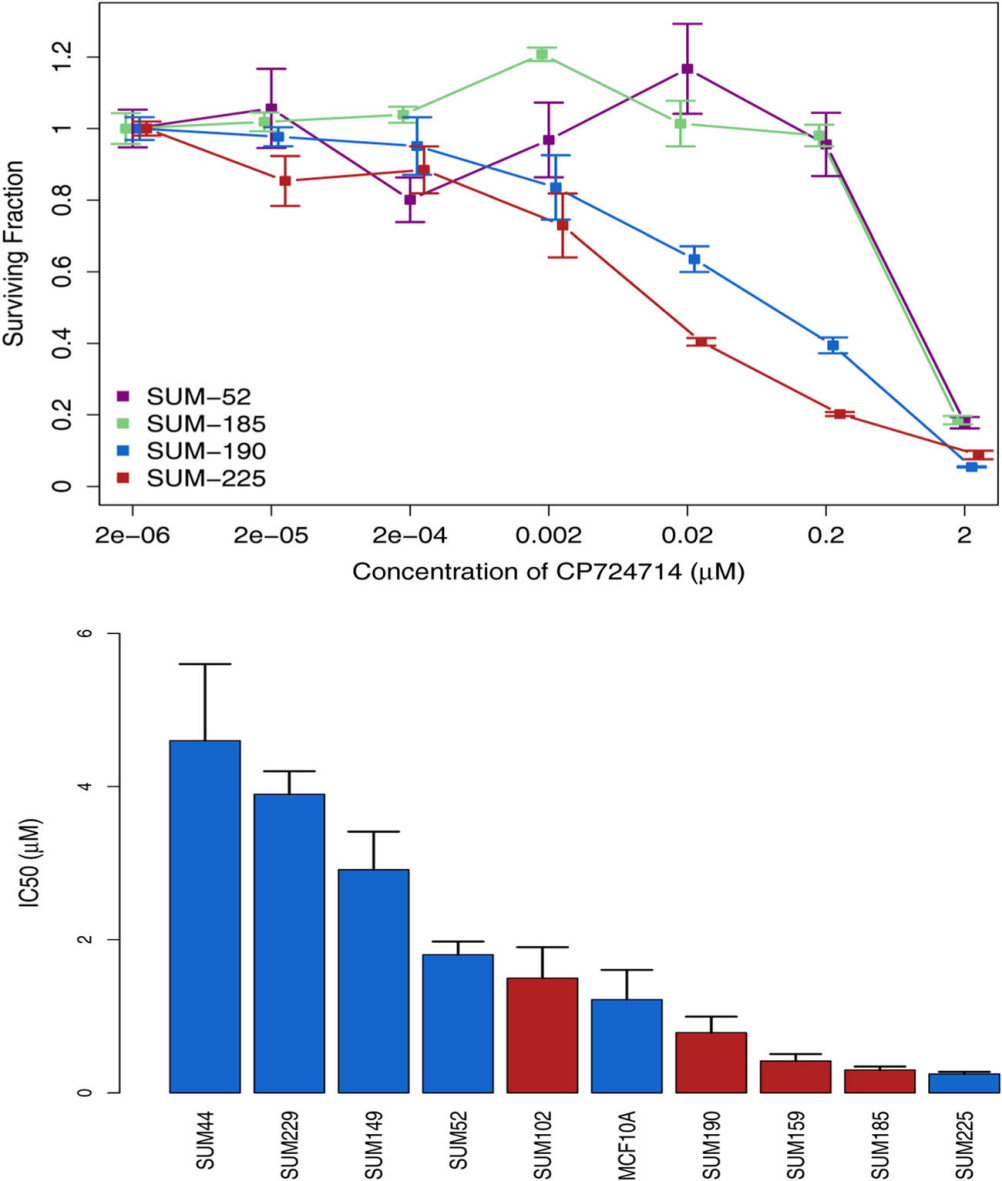
Oncogene signatures and drug sensitivity in SUM cell lines. **a** Relationship between surviving fraction and concentration of the HER2-specific tyrosine kinase inhibitor CP724714 for four SUM lines, each of which has an amplification and overexpression of an RTK oncogene. Both SUM-225 and SUM-190 have *HER2* amplifications with overexpression, SUM-52 has an amplification and overexpression of *FGFR2*, and SUM-185 has an amplification/overexpression of *FGFR3*. Cells were treated over a 72-h period with varying concentrations of drug and the number of viable cells per well was determined using the Celigo cell analyzer following staining with Hoechst stain for total cell number and propidium iodide to determine the number of dead cells. The surviving fraction was number of viable cells per well at 72 h divided by the number of cells per well before drug treatment. **b** SUM breast cancer cell lines were treated with varying concentrations of the class I alpha-specific PI3′K kinase inhibitor Alpelisib as described above and the IC_50_ concentrations for each cell line was determined using GraphPad 4.0 for all but two of the SUM lines. For the cells most resistant to the drug, we estimated the IC_50_s using a local polynomial regression (LOESS with a span = 1) to fit a local regression curve over the range of data and determine the IC_50_ value. The 95% prediction interval was calculated around the curve, and the interval at the point where cell concentration was predicted to be 50% of the starting value (coinciding with the IC_50_ concentration) was inverted to derive the error for the IC_50_ value.

One of the most powerful applications of genome-scale shRNA screens is the functionalization of genomic alteration data that are derived from sequencing or array-based applications. It is well-known that breast cancers, like most cancers, are genomically complex and that most of the genomic alterations that occur do not contribute directly to the malignant potential of the cells and are therefore poor drug targets. Thus, by combining data derived from essentiality screens with data derived from copy number analysis, gene expression analysis, and exome sequencing, one can quickly reduce the complexity of these data sets and identify the driving oncogenes in each cell line. We refer to the gene sets that are derived from such an analysis as the *oncogene signatures* for a given cell line, and we have reported on these for some of the SUM lines in previous publications^10,12^. We thus wanted to develop a tool that would allow anyone to ascertain the several types of oncogene signatures for any breast cancer cell line for which these genomic data sets are available, and this is now available on the SLKBase. By using the Oncogene Signature Tool, one can choose a breast cancer cell line and immediately identify three types of oncogene signatures. The first is the Candidate Oncogene Signature, which comprises genes that are genomically altered in the cell line, and are considered to be bona fide human oncogenes as indicated in the OncoKB database^27^. Thus, this list shows all *candidate* oncogenes that are genomically altered in the cell line, and their score in the shRNA screen provides information on the essentiality of each altered gene in the cell line. The second gene set is the Overall Oncogene Signature, which comprises any gene that is genomically altered in the cell line of interest that was also a hit in the functional screen, regardless of whether they are considered bone fide oncogenes. Thus, any gene that is genomically altered, by either gene amplification or point mutation, and was a hit in the functional screen is reported along with the expression level of the gene and its potential druggability. Finally, the Functional Oncogene Signature is the synthesis of the first two gene sets and shows the genes that are genomically altered, considered to be bona fide oncogenes, and were hits in the functional screen, along with their expression levels and druggability. The Candidate Oncogene Signature for the SUM-185 breast cancer cell line is shown in Table 1. The Overall and Functional Oncogene Signatures for this cell line are shown in Supplementary Tables 1 and 2. As can be seen, for each gene in each table, data on copy number, mutation status, and screen hit status are presented, as well as any existing drugs that target those oncogenes. The functional oncogene signature for the SUM-185 cell line is particularly interesting and shows that these cells have three functional and druggable oncogenes (*FGFR3, PIK3CA,* and *BCL2L1*), and we have shown previously that targeting these oncogenes using low doses of appropriate targeted drugs yields dramatic synergistic and cell line-specific lethality^12^. The Candidate Oncogene Signature for the SUM-190 cell line is shown in Table 2 and the other oncogene signatures for this cell line are shown in Supplementary Tables 3 and 4. The oncogene signatures for this cell line highlight the importance of having three separate gene lists. As we^12,28,29^ and others have published^30–35^, the SUM-190 cell line, derived from a patient with inflammatory breast cancer, has a *HER2* gene amplification, and HER2 is overexpressed at the mRNA and protein level. And yet, HER2 was not a hit in the functional screen for these cells. This is contrast to the SUM-225 cells, which also has *HER2* amplification^28,29^ and for which HER2 was a hit in the functional screen (Supplementary Table 5). This suggests that HER2 is more essential for the SUM-225 cells than the SUM-190 cells, despite their similar amplification and overexpression of HER2. And while both cells are sensitive to the HER2-targeted drug CP724714 compared to non-HER2-amplified cell lines, SUM-190 cells are 10-fold less sensitive to the targeted drug than are SUM-225 cells, a finding in keeping with the screen data (Fig. 1a)^26^. By contrast, the SUM-52 and SUM-185 cell lines, which have amplifications and overexpression of *FGFR2* and *FGFR3*, respectively, were as expected, highly resistant to the HER2-targeted drug. The SUM-190 Oncogene Signatures also shows that these cells have a commonly observed point mutation in the PIK3CA oncogene, and this gene was a strong hit in the functional screen. Figure 1b shows the IC_50_ values for the Class I alpha-specific PI3′Kinase drug Alpelisib for the SUM-line panel and shows that the SUM-190 cells, as well as the other SUM lines with *PIK3CA* point mutations (red bars in the figure) are highly sensitive to this drug. Thus, in SUM-190 cells, for which HER2 lies upstream of PI3′Kinase signaling, PIK3CA is a better druggable target than HER2, a result predicted by the shRNA screen data. Interestingly, the *HER2*-amplified SUM-225 cell line is also highly sensitive to Alpelisib, an observation made with other *HER2*-amplified breast cancer cell lines, as can be observed using the Functional-Druggable Target tool on the SLKBase. The concentration–response curves for Alpelisib across the SUM cell line panel are shown in Supplementary Fig. 1. The oncogene signatures for the other SUM lines and 25 other commonly used breast cancer cell lines can be viewed directly on the SLKBase.

**Table 1:** Candidate Oncogene for SUM-185 cells.

Gene symbol	QuantLog	QuantLogRank	ScreenHit	LogFoldChange	DnaAmp	Mutation	Occurences in cosmic	Existing drugs
*BCL2L1*	5.8	5	1	1.16583842	1.1286		0	Obatoclax Mesylate, Navitoclax, TW 37
*FGFR3*	2.75	261	1	2.87313456	1.1797		0	PD173074
*PIK3CA*	2.45	579	1			PIK3CAp.H1047R	1889	ZSTK474, PI-103, A66, BKM120
*ASXL1*	2.16	1366	0	0.49973277	1.1286		0	
*TP53*	1.93	2785	0	−0.9858457		TP53p.Q144*	47	
*PPP2R1A*	1.74	4937	0	0.42676332	0.8502		0	
*UPF1*	1.7	5503	0	1.3309692	0.9679		0	
*WHSC1*	1.46	9995	0	0.09894565	1.1797		0

**Table 2:** Candidate Oncogene Signature for SUM-190 cells.

Gene symbol	QuantLog	QuantLogRank	Screen hit	Log fold change	DnaAmp	Mutation	Occurences in cosmic	Existing drugs
*PIK3CA*	23.63	88	1			PIK3CAp.H1047R	1889	ZSTK474, PI-103, A66, BKM120
*EPHA5*	8.05	787	1	0	1.3009		0	
*CRKL*	5.26	2135	0	2.08183288	3.3127		0	
*CD274*	4.8	2621	0	0.81917265		CD274p.R260C	1	
*ERBB2*	2.48	8269	0	3.48529258	4.4293		0	Lapatinib, CP724714, CUDC-101, Afatinib
*CCND1*	2.4	8613	0	1.84218813	1.2862		0	
*PAK1*	2.02	10371	0	1.19466668	1.0625		0	IPA-3
*NBN*	1.9	11023	0	0.74382708	0.8672		0	
*EED*	1.82	11397	0		1.3939		0	
*FGF4*	1.65	12158	0	0.04983281	1.2862		0	
*FGF3*	1.6	12404	0		1.2862		0	
*CREBBP*	1.36	13493	0	1.33574903	0.8509		0	
*FGF19*	1.28	13826	0	−0.28420775	1.2862		0	
*FAM58A*	1.19	14135	0	1.23522966	0.9519		0	
*BRCA1*	0.95	14857	0	1.7257694	3.0084		0	
*RAD51L3*	0.76	15134	0	0.21738458	1.1823		0

The concept of oncogene addiction predicts that targeting functional-druggable oncogenes has a profound and specific effect on proliferation and/or survival in cells with those driving oncogenes. The oncogene signatures for the SUM lines make predictions about sensitivity to oncogene-targeted drugs, and these predictions are borne out by drug sensitivity data, as shown in Fig. 1 for HER2 and PIK3CA-targeted drugs, and as we have published for FGFR2, FGFR3, and BCL2L1 (ref. ^12^). Thus, elucidating the oncogene signatures of breast cancer cell lines is the starting point for developing targeted drug strategies that will be highly effective when used at low concentrations, and thus yield a high therapeutic index.

### Functional-druggable signatures

**Fig. 2:**
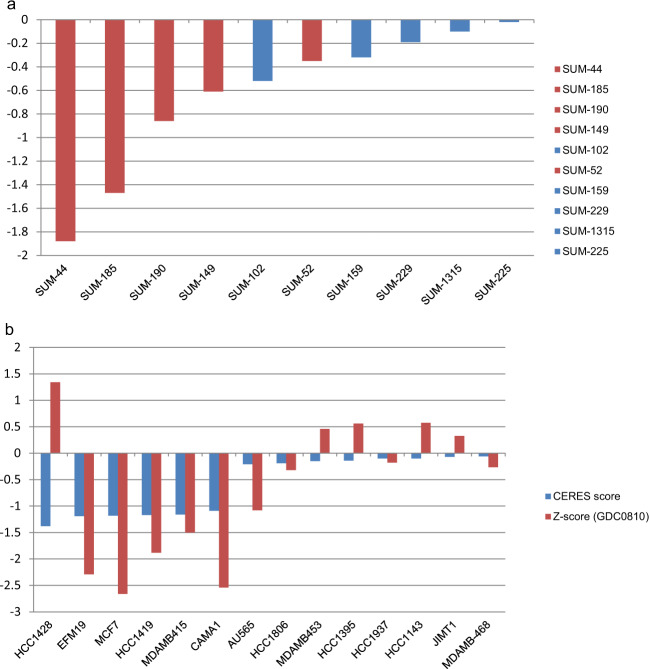
Relationship between gene essentiality and drug sensitivity in breast cancer cell lines. **a***Z*-scores (a measure of drug sensitivity) for the drug Navitoclax across the panel of SUM breast cancer cell lines. The *Z*-scores for each cell line treated with Navitoclax was calculated by subtracting the IC_50_ for each cell line from the geometric mean IC_50_ for this drug across the panel of 876 cancer cell lines and dividing by the standard deviation of the mean. Negative *Z*-score values indicate cells that are more sensitive (have lower IC_50_s) than the geometric mean IC_50_ across the cell line panel. The *Z*-score value indicates how many standard deviations from the mean the IC_50_ is for each cell line. Cells are considered highly sensitive to the drug, with respect to all other cell lines, when the *Z*-score is ≤−1.5. **b***Z*-scores and CERES scores for 14 breast cancer cell lines that were treated with the ER degrader GDC0810 and part of the Achilles CRISPR gene essentiality screen. Data from this figure were derived from our MySQL database after downloading data from DepMap portal. As indicated above, *Z*-scores ≤ −1.5 indicate sensitivity to the drug, and a CERES score of <−0.5 indicate hits in the screen.

One of the advantages of genome-scale shRNA screens is that they identify a large number of essential genes, most of which are not genomically altered, and, therefore, not bona fide oncogenes. And yet, because these genes are essential to the growth and/or survival of the cells, they can be good drug targets as well. In order to fully leverage the data derived from our genome-scale functional screen, we created the Functional-Druggable Signature tool, which merges the shRNA or CRISPR screen hit data with data derived from the Genomics of Drug Sensitivity in Cancer database (https://www.cancerrxgene.org)^36^. This database lists all of the targeted drugs that have been tested experimentally against a panel of nearly 1000 cancer cell lines. The functional-druggable signature tool returns a list for each breast cancer cell line of genes that are both essential as determined by their hit status in the screen and druggable using a targeted agent. The functional-druggable signatures for three of the SUM lines are shown in Table 3, and the functional-druggable signatures for all the SUM lines, and for 27 other breast cancer cell lines, can be viewed on the SLKBase. The hypothesis that emerges from this analysis is that essentialness as determined by the screen hit data predicts drug sensitivity. We have performed preliminary experiments to examine this hypothesis using drugs that target BCL2L2/BCL2 (Navitoclax), and the results are consistent with this hypothesis. The *Z*-scores for this drug in each of the SUM lines are shown in Fig. 2a^26^ and the red bars indicate the cell lines for which either BCL2L2 or BCL2 (SUM-44) was a hit in the screen, and shows a good association between drug sensitivity and essentialness as determined in the screen. The concentration–response curves used to calculate the IC_50_ concentrations for Navitoclax for each of the SUM breast cancer cell lines are shown in Supplementary Fig. 2.

**Table 3:** Functional Druggable Signatures for three SUM cell lines.

SUM-44	Existing drugs
KIF11	*S*-Trityl-l-cysteine, Ispinesib Mesylate
MAP4K2	NG-25
CDK6	AT-7519, Palbociclib
BCL2	Obatoclax Mesylate, Navitoclax, TW 37
PIK3CD	Idelalisib

SUM-229	Existing drugs
EDNRA	Zibotentan
LCK	A-770041, WH-4-023, JW-7-24-1
DHX9	YK-4-279
MAPK11	TAK-715
CAPN1	MG-132
XIAP	Embelin

SUM-225	Existing drugs
TOP1	Camptothecin, SN-38
CHUK	GSK319347A, BMS-345541
FRAP1	Rapamycin, JW-7-52-1, Omipalisib, OSI-027, Temsirolimus, Dactolisib, AZD8055, QL-VIII-58
DHX9	YK-4-279
PRKCD	Midostaurin, XMD11-85h
KIF11	*S*-Trityl-l-cysteine, Ispinesib Mesylate
NR1H2	T0901317
HDAC3	Entinostat
EDNRA	Zibotentan
RAC1	EHT-1864
PLK1	BI-2536, GW843682X
BRD4	JQ1, I-BET-762, PFI-1
ERBB2	Lapatinib, CP724714, CUDC-101, Afatinib
PIK3CA	A66, BKM120

To begin to test this hypothesis more rigorously, we developed a version of the functional-druggable signature tool for the 27 additional breast cancer cell lines for which there are both functional genomic data from Project Achilles, and drug sensitivity data from the Genomics of Drug Sensitivity in Cancer database. Using this tool, one can directly examine the relationship between essentiality as determined by the screen hit score (CERES score) and the *Z*-score for each drug in each cell line, which is a measure of relative sensitivity for each drug across a panel of over 800 cell lines. An example of this is shown in Fig. 2b for the ER degrader GDC0810. With the exception of a single cell line, this result shows a strong association between essentiality, as determined in the screen, and drug sensitivity as indicated by the *Z*-score for the drug (A *Z*-score of <−2.0 indicates that the IC_50_ for this drug in this cell line is greater than 2 standard deviations less than the geometric mean IC_50_ for the entire panel). Work currently in progress is aimed at performing a rigorous statistical analysis of the association between essentiality and drug sensitivity for all targeted drugs across the entire cell line panel.

### KEGG Pathway Engine and Pathway Essentialness tool

**Fig. 3:**
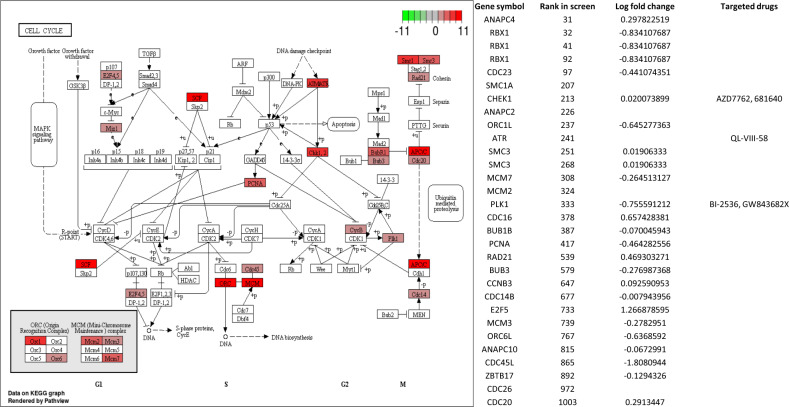
KEGG pathway engine analysis of the cell cycle pathway for SUM-149 cells. Results returned by the KEGG Pathway Engine for the essentialness of the Cell Cycle KEGG pathway in the SUM-149 cell line based on the screen hit data for that line. The KEGG Pathway Engine returns the pathway map with hit genes highlighted in red, and a table that shows for each hit gene, the rank in the screen, the expression level relative to normal cells as a Log2 ratio of the fold difference with respect to normal cells, and any targeted drugs associated with essential genes in the pathway.

**Fig. 4:**
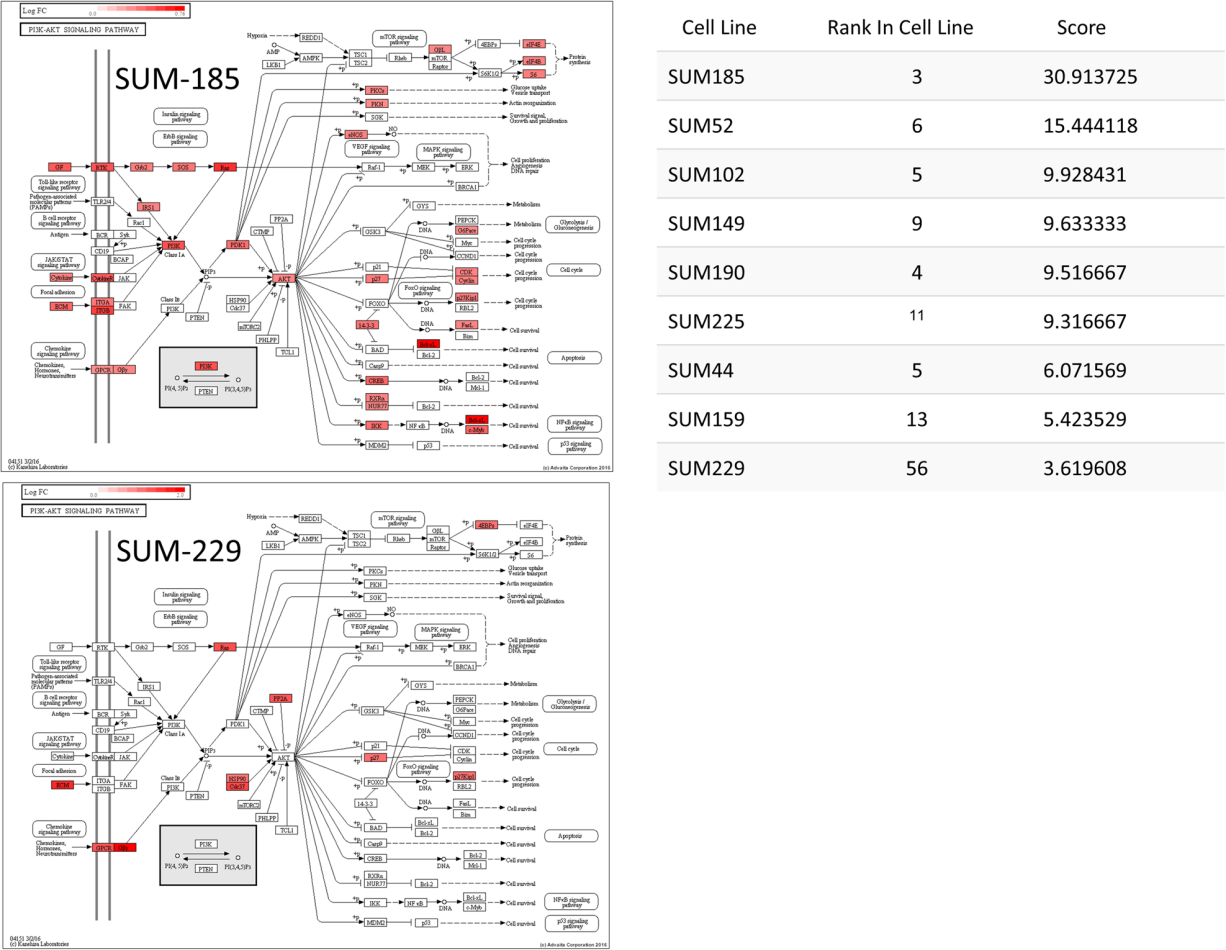
Variation in essentialness of the PI3′Kinase–AKT pathway across the SUM line panel. Screen hit data mapped to the PI3′Kinase/AKT KEGG pathway for SUM-185 and SUM-229 cells showing the wide variation in essentialness for this pathway across these two cell lines. Screen hit data were mapped to the pathway using the KEGG Pathway Engine. The right panel shows the relative essentialness of this pathway for the SUM cell line panel. The values were derived using the KEGG Pathway Analysis tool.

Another way to gain insight into the biology of human breast cancer cell lines is to use the screen hit data to identify the most important biological pathways for each cell line. To accomplish this goal, we developed the KEGG Pathway Engine, which maps the genes that were hits in the functional screen onto KEGG pathways. The KEGG Pathway Engine allows users to pick any KEGG pathway and determine its importance in any cell line in the database, obtain a visual picture of the pathway of interest with screen hits in the pathway displayed, and view the data associated with each hit gene in the pathway. A separate feature of this tool is the ability to view gene expression data for the specific cell line and pathway and compare that to the screen hit data. Figure 3 shows the results of mapping the screen hit data for the SUM-149 cells onto the Cell Cycle KEGG pathway, and indicates the genes in this pathway that were screen hits, with red color intensity being related to the strength of the screen hit, or hit rank. The right panel of the figure shows the screen hit data that are presented with the pathway map, along with the rank of each hit gene in the screen, its expression level, and any targeted drug for each screen hit gene. Figure 4 shows the results of the analysis of the essentialness of PI3K–AKT signaling in two SUM lines with the highest and lowest essentialness of this pathway, SUM-185 and SUM-229, respectively. As is shown in the figure, the SUM-185 cell line, for which *FGFR3* is a functional-driving oncogene activated by gene amplification, is highly dependent on this pathway for proliferation and survival. By contrast, other cell lines are less dependent on this pathway, and indeed, the SUM-229 cell line (lower left panel) has little or no reliance on this pathway for proliferation or survival as indicated by the small number of screen hit genes that map to the pathway.

**Fig. 5:**
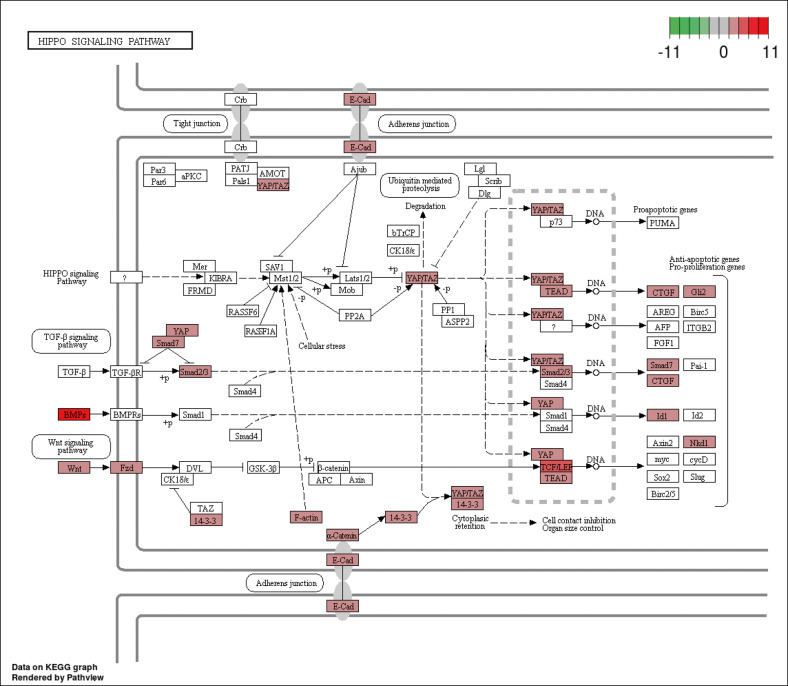
Essentialness of the HIPPO pathway in SUM-185 cells. KEGG Pathway Engine generated data showing the essentialness of the Hippo KEGG pathway to the SUM-185 cells as predicted by the KEGG Pathway Analysis tool.

**Fig. 6:**
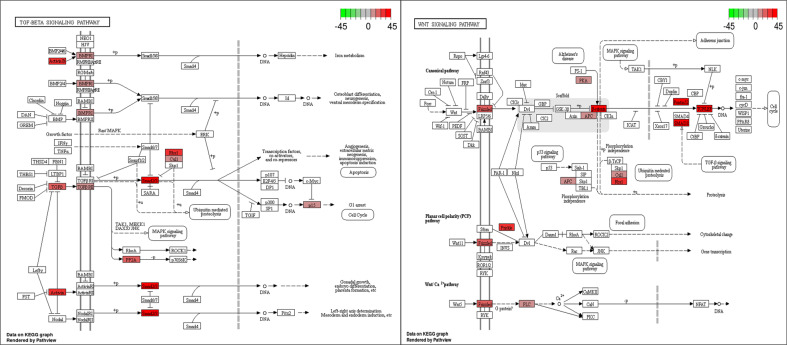
Essentialness of the TGF-beta and WNT signaling pathways in SUM-229 cells. KEGG Pathway Engine generated data showing the essentialness of the TGF-beta and WNT signaling pathways in the SUM-229 cells.

To increase to power of the KEGG pathway engine, we developed a Pathway Analysis tool that calculates the relative essentialness of all KEGG pathways and rank-orders them based on the level of enrichment of the screen hit data to the pathway. The algorithm also takes into account the proportion of the genes in the pathway that were screen hits, as well as the relationship of hit genes in the pathway to each other, such that screen-hit genes that are directly linked to other screen hits in the pathway receive more weight. Table 4 shows the top ten KEGG pathways by essentialness scores for the SUM-52 and SUM-229 cell lines. Using the Pathway Analysis tool, one can pick any cell line and obtain a rank-ordered list of essential pathways, as just described. Alternatively, one can choose a specific pathway and receive a rank ordered list of cell lines by their reliance on that pathway. Table 5 shows the rank order of the cell lines for essentialness of the transforming growth factor-β (TGF-β) and Hippo signaling pathways. This table shows that TGF-β signaling is highly essential for SUM-229 and SUM-1315 cells, and that Hippo signaling is highly essential for SUM-185 cells. Figure 5 shows the SUM-185 screen hits that map to the Hippo pathway, and shows significant enrichment for genes in this pathway, including the key transcription factors and target genes that define this pathway. By contrast, for SUM-229 cells and SUM-1315 cells, the TGF-β and WNT signaling pathways are highly essential, as indicated by their scores and the connectedness of hit genes in these pathways (Fig. 6a, b). This is interesting because the SUM-229 line is a *KRAS*-transformed cell line that falls into the basal/claudin-low subset of triple-negative breast cancers and has been predicted by others to be enriched for expression of genes in the TGF-beta pathway^37–40^. Thus, the Pathway Engine result is not only consistent with this prediction but also identifies the specific genes within the pathway that are most essential for these cells. The WNT signaling and TGF-beta signaling pathways were also found to be important in SUM-1315 and SUM-159 cells, the latter of which is also a *MYC/HRAS* transformed cell line. Thus, the KEGG pathway engine and Pathway Analysis tools, coupled with the functional-druggable signature tool, help to identify strategies and drug targets even in breast cancer cells that do not express functional-druggable oncogenes.

**Table 4:** Top 10 Essential KEGG pathways for SUM-52 cells.

	Score	# hits	Proportion of hits	Rank
Pathways in cancer—*Homo sapiens* (human)	13.283333	36	0.159292	1
RNA transport—*Homo sapiens* (human)	12.416667	32	0.3232323	2
PI3K–Akt signaling pathway—*Homo sapiens* (human)	10.75	22	0.2588235	3
Cell cycle—*Homo sapiens* (human)	9.166667	18	0.225	4
Human papillomavirus infection—*Homo sapiens* (human)	8.7	26	0.2131148	5
Hepatocellular carcinoma—*Homo sapiens* (human)	7.97619	17	0.2328767	6
Proteoglycans in cancer—*Homo sapiens* (human)	7.533333	17	0.136	7
Autophagy—animal—*Homo sapiens* (human)	7.333333	16	0.183908	8
Insulin signaling pathway—*Homo sapiens* (human)	7.083333	13	0.2096774	9
Human T cell leukemia virus 1 infection—*Homo sapiens* (human)	6.866667	21	0.1826087	10

**Table 5:** Rank order of essentialness of TGF-beta and Hippo signaling pathways in SUM lines.

TGF-beta signaling			Hippo signaling		
Cell line	Rank in cell line	Score	Cell line	Rank in cell line	Score
SUM229	19	5.5108696	SUM185	57	11.896766
SUM185	168	5.2572464	SUM229	7	7.06592
SUM225	95	3.3913043	SUM52	60	5.159204
SUM149	96	2.4492754	SUM225	60	4.742537
SUM52	165	1.6101449	SUM159	19	3.788557
SUM159	99	1.1956522	SUM102	16	3.655224
SUM190	131	1.1811594	SUM149	84	2.657534
SUM44	169	0.5289855	SUM44	30	2.6242
SUM102	128	0.5289855	SUM190	65	2.370647

### Gene Query tool

The data mining tools described above are powerful ways to analyze gene lists and derive biological significance from them. We also wanted to build a tool that would allow researchers to rapidly and easily obtain information on individual genes for any cell line in the database. For this, we developed the Gene Query tool, which allows users to search for any annotated gene in the genome and obtain functional genomic and druggability for that gene in any cell line. As an example, the results of a query for the *BCL2L1* gene, which is of developing interest as a therapeutic target in breast cancer, are shown in Table 6. The search results show that *BCL2L1* is amplified, overexpressed, and a hit in the screen in SUM-185 cells. This gene, which is expressed at relatively normal levels in SUM-52, SUM-190, and SUM-149 cells, was a hit in the screen in these cell lines as well, and they are, indeed, sensitive to the targeted drug Navitoclax (Fig. 1b). A similar search for a related gene, *BCL2*, shows that it is overexpressed in SUM-44 cells, as would be expected for an estrogen-receptor-positive cell line, and was a strong hit in the SUM-44 screen. Thus, SUM-44 cells are also highly sensitive to Navitoclax with an IC_50_ of 0.2 µM, as this drug targets both BCL2 and BCL2L1. Thus, this gene query tool returns genomic, functional, and druggable data for any gene in any cell line with a simple mouse click.

**Table 6:** Gene Query Results for BCL2L1.

Cell line	Rank in screen	ScreenHit	LogFoldChange	DnaAmp
SUM185	5	1	1.165838415	1.1286
SUM52	339	1	0.531558612	
SUM190	598	1	0.351049571	
SUM149	1043	1	0.273146406	
MCF10A	1283	0		
SUM1315	1561	0		
SUM229	4105	0	7.609930667	
MCF7_LTED	5051	0		
MCF7	5976	0		
SUM44	9494	0	−0.008303944	
SUM225	9547	0	0.714527433	
SUM102	12,000	0	0	
SUM159	13,674	0	6.070714333	

### Proteomics tool

**Fig. 7:**
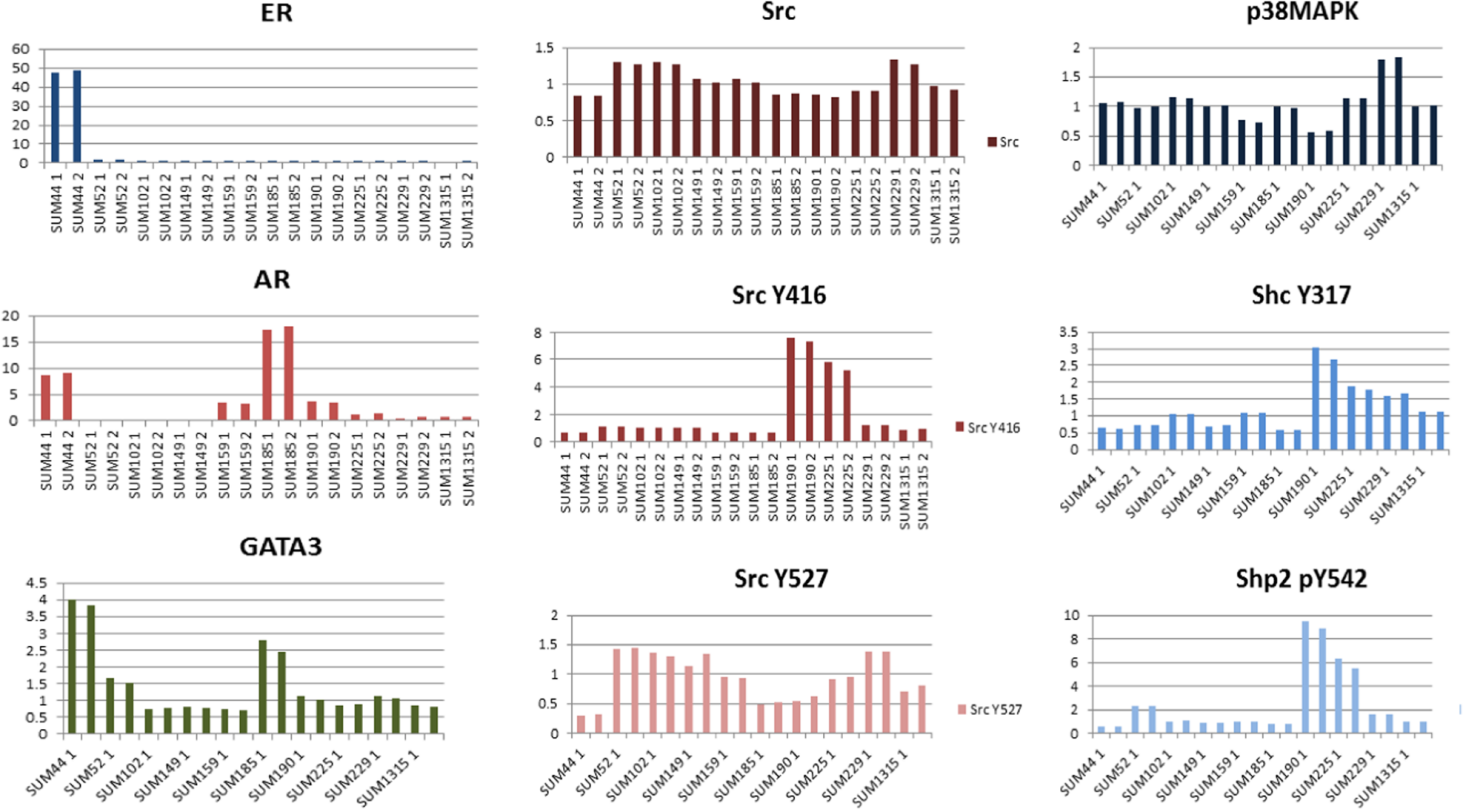
Proteomic and phospho-proteomic analysis of the SUM panel. **a** Relative expression values for various cell signaling proteins and phospho-proteins that were determined for the SUM line panel using Reverse Phase Protein Arrays (RPPA).

To make the data sets in the Knowledge Base more complete, we recently added proteomics data derived from RPPA analysis of the SUM panel of cell lines. The proteomics data are presented in two ways on the SLKBase. First, bar graphs are presented that show the data for a subset of proteins and phospho-proteins the expression of which varied widely across the panel (Fig. 7)^26^. Figure 7 shows that SUM-44 cells express very high levels of the estrogen-receptor (ER). In keeping with their ER expression, these cells also express high levels of GATA3. This figure also shows that androgen receptor expression varies widely across the cell lines with SUM-185 cells expressing the highest levels, as has been reported by others, and these cells also express high levels of GATA3. Figure 7 also shows the relative expression of key signaling molecules, including Src and phospho-Src. This analysis showed that although the expression level of c-Src protein itself is relatively constant across the cell line panel, SUM-225 and SUM-190 cells exhibit the highest levels of Src pY416, indicating a high level of Src kinase activity in these two HER2-amplified breast cancer cell lines. The relative protein expression of CyclinD1, CyclinD2, and GAB2 proteins across the cell line panel is shown in Supplementary Fig. 3. This is of interest because both *CCND1* and *GAB2* are present in the 11q14 genomic region, which is amplified in SUM-44 and SUM-190 cells. And, while CyclinD1 protein levels did not vary significantly across the panel, GAB2 protein is highly overexpressed in SUM-44 cells and in SUM-190 cells. This suggests that *GAB2*, and not *CCND1*, is the important driver gene from this amplicon in these cell lines.

To provide access to all of the proteomics data, we created the Proteomics Query tool, which allows a search for any protein or phospho-protein that was measured by RPPA and returns normalized linear expression data for all proteins measured on the array rank-ordered by expression levels in the cell lines. This tool can be used in conjunction with the Gene Query tool to rapidly compare expression of a number of important genes at both the mRNA and protein levels.

### Supporting cell line information on the SLKBase

One of the original goals of the SLKBase was to provide an online information resource for the SUM breast cancer cell lines for investigators around the world who make use of these cells. Thus, in addition to the data mining tools that form the core of the SLKBase, each SUM breast cancer cell line has its own home page containing information on the patient from which the cell line was derived, a summary of the molecular characteristics of each line, and a bibliography of published papers containing data derived from each cell line. This information is designed to be used in conjunction with the data derived using the data mining tools to allow researchers to have a complete biological picture of each model cell line. It is important to keep in mind that every breast cancer cell line was derived from a breast cancer patient, and the cell line, like the patient, is complex and is more than just a single feature to be studied in a reductionist manner.

### Expansion of the SLKBase to all breast cancer cell lines

As indicated in the preceding pages, the SLKBase was originally designed as a resource for users of the SUM breast cancer cell lines. However, other laboratories have developed similar omics and functional screen data sets for other commonly used breast cancer cell lines, and those data have been deposited in publicly available repositories. Thus, we have recently incorporated some of these data sets into our MySQL database and modified our data mining tools so they can be used with any breast cancer cell line for which reliable omic and functional data are available. Thus, the SLKBase now contains new pages with drop down menus for commonly used breast cancer cell lines, which dramatically increases the power of this resource.

## Discussion

Breast cancer cell lines have been a focal point of breast cancer research for over 50 years, and thousands of papers have been published that make use of these models. Despite that, what is sometimes lost in the discussion about the use of breast cancer cell lines in research is the connection between the line itself, and the patient from which it was derived. As a result, most investigators who use these model systems do so because they have a specific phenotype or specific set of genes that are of interest to the researcher. Thus, MCF-7 and T47D cells have been widely used to study the roles of the estrogen and progesterone receptors in breast cancer. SKBR3 and BT-474 cells have been widely used by investigators interested in *HER2* amplification and overexpression in breast cancer, and MDA-MB-468 cells have been often used to study EGFR signaling in breast cancer. And while these studies have proven fruitful, there is a sense of diminishing returns for this type of research when it comes to human cell lines, and this has sparked efforts to use alternative models, such as PDX and organoid cultures, to study further the biology of human breast cancer.

Rather than considering the use of breast cancer cell lines because of one or two specific characteristics, it is now possible to consider cell lines as individual breast cancer patients, and use functional genomics to identify, in an unbiased manner, the most important genes, pathways, and druggable targets for each line. Such a systems-level approach can shed new light on many breast cancer cell lines. For example, the first line that we developed in our laboratory was SUM-44PE^41^. These cells were derived from the pleural effusion metastasis of a patient with estrogen-receptor-positive lobular breast cancer, and indeed, these cells, like MCF-7 cells, can be used to study the role of the estrogen receptor in the biology of breast cancer^42–47^. However, by performing the type of systems-level functional genomic analysis provided on the SLKBase, one can see that SUM-44 cells exhibit amplification of the 8p11–p12 genomic region, which we and others have published on, and data from the shRNA screen identify *KAT6A* (Myst3) and *EIF4EBP1* as important driver genes from this amplicon in these cells^10,44,48^. In addition, SUM-44 cells overexpress BCL2, which was a strong hit in the screen, and the cells are exquisitely sensitive to Navitoclax and Venetoclax. *CDK6* was also a strong hit in the shRNA screen in SUM-44 cells, and these cells are sensitive to palbociclib, as predicted from the functional druggable signature for these cells. By exploring important KEGG pathways, one can see that SUM-44 cells express a number of cytokines and chemokines, many of which, such as CCL1, LIFR, CCL25, and others, were strong hits in the functional screen, indicating their importance in the proliferation and survival of these breast cancer cells. Finally, these cells, having been derived from a patient with lobular breast cancer, have a point mutation in the *CDH1* gene rendering them E-cadherin null, and thus, a good model of lobular breast cancer^47^. These findings demonstrate that overexpression and activation of the ER is just one feature of the SUM-44 cell line, and this phenotype occurs in the context of other molecular and cellular features important to the biology of the cells and the patient from whom they came. Thus, the SLKBase can be used to explore the biology of individual breast cancer cell lines, which yields novel and important observations on breast cancer cell lines that can be used to elucidate new strategies for reverse engineering of human breast cancer cells.

Oncogene addiction^49–51^ is the primary driving principle behind the orthogonal omics strategy that is key to the SLKBase. The principle of oncogene addiction proposes that cancer cells have one or more driving oncogenes to which the cells are addicted for their proliferation and survival, and the correctness of this principle has been shown in many laboratory and clinical studies. Based on this principle, we predicted that we could identify the functional-driving oncogenes in any cell line by an orthogonal analysis of gene amplification, gene expression and point mutation data, with shRNA screen data, which shows all of the genes in a given cell line that are essential for their growth and survival. Furthermore, the principle of oncogene addiction predicts that, for those functional-driving oncogenes that are druggable, cells should be exquisitely sensitive to drugs that target the products of those driving oncogenes. The results of our studies with the SUM lines support the predictions made by the principle of oncogene addiction.

A second driving principle that underlies our approach to functional genomics is that since screen hits identify genes essential for growth and/or survival of the cancer cells, cells that express essential genes that are druggable should be sensitive to drugs that target those genes. For this reason, the SLKBase not only provides data on functional oncogenes in each breast cancer cell line, it also provides functional-druggable signatures for each cell line. Indeed, results of our experiments support the connection between essentialness as determined in the shRNA screens and drug sensitivity for a number of targeted drugs such as EGFR inhibitors, BCL2/BCLXL inhibitors, p38 MAP kinase inhibitors, PLK1 inhibitors, palbociclib, and others. In addition, our KEGG pathway analysis tools help to solidify the connection between functional druggable targets and essential pathways in individual cancer cell lines. The recent paper by Lin et al.^52^ highlights the importance of linking gene essentiality data with drug sensitivity for the proper clinical development of targeted drugs.

One concern that investigators have with genome-wide screens is the possibility of false-positive results that point to genes that are incorrectly identified as essential. In our hands, using the Cellecta library of shRNAs and the statistical method we developed for analyzing the screen data, false positives have not been a significant issue. So far, we have never failed to confirm a screen hit using individual shRNA constructs that target putative essential genes. However, we do consider false negatives to be an issue with our screen data. The cut points that we chose to determine the genes considered to be hits in the screen typically yield approximately 1000 hits per cell line. We intentionally chose a conservative cut point so as to minimize false positive hits. The consequence of this decision is more false-negative results that one has to be cautions of. For example, SUM-159 cells have a classic *HRAS* point mutation, and in the SUM-159 screen, this gene was ranked 2378 out of approximately 15,000 genes queried in the screen. Had we chosen a more liberal cut-off point; this gene could have been considered a hit. For this reason, in all of the data that are returned on specific genes using the data mining tools in the SLKBase, the actual screen hit ranking is provided along with the hit status, so investigators can see for themselves how “essential” any gene is in any given cell line. We have identified other examples of false negatives for the SUM lines that are important to discuss. For example, SUM-44 cells express very high levels of ESR1 mRNA and ER protein, both of which can be seen on the SLKBase. Furthermore, we have previously shown that while SUM-44 cells are relatively estrogen-independent and Tamoxifen-resistant, these cells are sensitive to ER degraders such as fulvestrant, and knock-down of ESR1 using shRNAs resulted in profound loss of cell viability. Thus, the screen hit ranking of ESR1 of 4857 in SUM-44 cells appears to be a false-negative result and suggests that the shRNAs that target ESR1 in the Cellecta library did not have a strong enough effect on knock-down of the very high mRNA levels to result in a sufficiently high drop-out rate to score as a hit in the screen.

In summary, our in-depth analysis of the SUM breast cancer cell lines, and more recently, other breast cancer cell lines, reinforces the importance of taking a systems-level approach to understanding breast cancer cell lines, and not lose sight of the fact that each cell line was derived from an individual patient with a specific set of molecular characteristics. As part of this approach, it is critical that investigators obtain and work with cancer cell lines appropriately to ensure that they are working with the cells they think they are, and to ensure that phenotypic drift is minimized, as any alteration in culture conditions from those originally used to develop the cell line can result in selection of subpopulations present within the cell line. By taking such measures, cancer cell lines are stable models of the type of breast cancer that was experienced by the patients from which they came, making it possible to use these model systems to develop novel and innovative reverse-engineering strategies for each cell line (patient) and ultimately use those strategies to solve the *n* of 1 problem, and truly make targeted cancer therapy precise and effective.

## Methods

### Regents and cell lines

All inhibitors were purchased from Selleckchem. The SUM breast cancer cell lines were maintained as described previously. MCF10A cells were a gift from Dr. Herb Soule at the Michigan Cancer Foundation. The molecular subtypes of each of the SUM lines along with additional information regarding each line are presented in the SLKBase (https://sumlineknowledgebase.com/?page_id=350). Briefly, SUM-44 and SUM-52 are luminal B cells; SUM-102, SUM-149, SUM-159, SUM-229, and SUM-1315 are triple-negative breast cancer cells. More specifically, SUM-159 and SUM-1315 cells map to the claudin-low subtype. SUM-190 and SUM-225 cells are HER2-positive breast cancer cells, and SUM-185 maps to the androgen-receptor enriched sup-type of breast cancer cells.

### Small-molecule inhibitor dose response assays

Cells were plated in 24-well plates at a density of 15–30,000 cells per well. Cells were allowed to recover for 4 days before being treated in triplicate with the indicated inhibitors or DMSO control every 24 h for 4 days. On the fifty day, cell number was determined by harvesting and counting nuclei on a Z1 Coulter Counter (Beckman Coulter, Brea, CA, USA). To prepare nuclei for counting, cells were washed three times with PBS, incubated on a rocker table with 0.5 ml per well HEPES/MgCl_2_ buffer (0.01 mM HEPES and 0.015 mM MgCl_2_) for 5 min, and lysed for 10 min with ethyl hexadecyldimethylammonium solution. For most of the cell lines, IC_50_s and the standard deviations were determined using GraphPad. For the SUM-229 and SUM-44 cell lines that were resistant to Alpelisib and as a result did not yield a large enough change in cell growth to result in a sigmoid curve interpretable by GraphPad, a local polynomial regression was used (LOESS with a span = 1) to fit a local regression curve over the range of data and determine the IC_50_ value. The 95% prediction interval was calculated around the curve, and the interval at the point where cell concentration was predicted to be 50% of the starting value (coinciding with the IC_50_ concentration) was inverted to derive the error for the IC_50_ value.

### Comparative genomic hybridization

Microarrays with an average resolution of 35 kb (Agilent Human Genome CGH Microarray 44k chip) were hybridized after direct labeling of DNA with fluorescent dyes. DNA extraction was performed using standard column purification (Qiagen) and normal human female DNA was used as the reference. Dye-reversed replicates were performed. Regions of chromosomal amplification and deletion were determined based on circular binary segmentation provided by the Bioconductor DNA copy library.

### Expression profiling

Total RNA was prepared using standard methods. RNA integrity was verified on an Agilent 2200 TapeStation (Agilent Technologies, Palo Alto, CA) utilizing samples with RINs ≥8. Total RNA (100–200 ng) was used to prepare RNA-Seq libraries using the TruSeq RNA Sample Prep Kit following the protocol as described by the manufacturer (Illumina, San Diego, CA). Libraries were clustered at a concentration to ensure at least 100 million reads per sample on the cBot as described by the manufacturer (Illumina, San Diego, CA). Clustered RNA-seq libraries were paired-end sequenced using version 4 chemistry with 2 × 125 cycles on an Illumina HiSeq2500. Demultiplexing was performed utilizing bcl2fastq v2.17.1.14 to generate Fastq files for downstream analysis.

### RPPA analysis

For RPPA analysis, cells were lysed in 100 μl RPPA lysis buffer containing 1% Triton X‐100, 50 mM HEPES, 150 mM NaCl, 1.5 mM MgCl_2_, 1 mM EGTA, 100 mM sodium fluoride, 10 mM sodium pyrophosphate, 1 mM sodium orthovanadate, 10% glycerol and protease/phosphatase inhibitors (Roche #05056489001/04906837001). Protein concentrations were determined by Bradford assay (BioRad) and concentrations were adjusted to 1 mg/ml. The samples were then mixed with 4× SDS sample buffer containing 0.2 M Tris–HCl (pH 8.0), 40% glycerol, and 8% SDS, boiled for 5 min, and stored at −80 °C until shipment to the RPPA Core Facility at MD Anderson for analysis.

### Exome sequencing

Exome sequencing of SUM cell line DNA was performed essentially as described previously^53^. Briefly, Agilent Sure Select XT reagents were used to prepare sequencing libraries. Hybrid capture was performed using Agilent Sure SelectXT Human All Exon V4 + UTRs, and 100 bp paired-end sequencing was performed on a HighSeq2000 achieving a median coverage of greater than 50-fold. Reads were aligned to the human reference genome GRCh37 using the Burrow-Wheeler Aligner. The data were processed further using the Genome Analysis Toolkit (GATK). For inclusion in the SLKBase, we cross-referenced all called SNVs with data in COSMIC and only report mutations that have occurred in COSMIC > 5 times. For the data from DepMap portal, we only report on the SLKBase mutations considered to be hot-spot mutations in COSMIC.

### Genome-scale shRNA screens

The detailed methods that we used for our shRNA screens of the SUM lines have been reported previously^12^. Briefly, virus pools expressing shRNA constructs were prepared according to the Cellecta Pooled Lentiviral shRNA Libraries User Manual protocol (www.cellecta.com). HEK 293 T cells were transfected with each of the three Cellecta library plasmid DNA pools (Human Modules 1–3) and the Cellecta Ready-to-Use Packaging Mix (Cat #CPCP-K2A). For each module, virus was titered and used to transduce 5 × 10^7^ target cells at a MOI of ~0.3 in the presence of 5 µg/ml polybrene. Following transduction, cells were cultured for 3 days to allow expression of the resistance marker and non-transduced cells were eliminated from the culture by addition of the selective agent puromycin to the growth media. Three days after the addition of puromycin, cells were trypsinized and one half of the total population was harvested for genomic DNA preparation. The remaining cells were plated and grown for ~5–7 population doublings before harvesting for genomic DNA preparation. Genomic DNA was prepared by phenol:chloroform extraction according to the Cellecta Pooled Lentiviral shRNA Libraries User Manual protocol.

Barcode sequences were amplified from genomic DNA by two rounds of PCR as described previously. Amplified barcode sequences were run on a 3.5% agarose gel and purified using a QIAquick Gel Extraction Kit (Qiagen) according to the manufacturer’s instructions. Isolated barcode sequences were further purified using the PureLink Quick PCR Purification Kit (Invitrogen) according to the manufacturer’s instructions. For sequencing, purified barcodes were diluted to 0.75 ng/µl using buffer EB (Qiagen). Amplicons were clustered at 17 pM including 30% (v/v) PhiX to add sequence diversity. Single end (SE) clustering was performed on a Cbot according to the manufacturer’s protocol (Illumina, San Diego, CA). A total of 36 cycles of SE sequencing were performed on an Illumina HiScanSQ. Custom primer GexSeqS (5′-AGAGGTTCAGAGTTCTACAGTCCGAA-3′, HPLC Purified) was added to the Illumina sequencing primers at 0.5 µM. Fastq files were generated using CASAVA 1.8.2 and processed using Trimmomatic software (www.usadellab.org) to trim read lengths to 18 nucleotides. Trimmed reads were deconvoluted using a Cellecta Barcode Analyzer and Deconvoluter software. Fold-depletion scores for each shRNA were calculated as the ratio of the read count at the reference time point versus the final time point.

To identify screen hits, log-transformed depletion scores and a quantile estimation approach in which the 80th percentile for each gene was calculated from its empirical distribution were used. This avoided the bias induced by the varying number of scores per gene and accounted for the skewness of the empirical distributions. Genes were then ranked by this log-quantile score and the empirical distribution of the log-quantile score was calculated.

To generate a null distribution of log fold-depletion scores, it was assumed that the majority of genes (>95%) would not be depleted, and their log-quantile scores would have a normal distribution. Based on this assumption, the median of the empirical distribution was used as an estimate of the mean of the null distribution. The estimate of the standard deviation of the null distribution was defined as the 97.5th quantile minus the 2.5th quantile, divided by 4. This was based on the knowledge that 95% of the data in a normally distributed variable falls between ± two standard deviations from the mean. Using this null distribution, all genes having log fold-depletion scores that were larger than the 95th percentile of the null distribution were identified as “hits”. Using this method, all genes that were hits in the screen had at least two, and usually more, shRNAs with depletion scores above the cut point.

### Acquisition and modification of data for other breast cancer cells

We acquired data derived from other breast cancer cell lines from the Broad Institute’s Cancer Dependency Map project (DepMap)^8^, available at https://depmap.org/portal/download. We obtained metadata for each breast cancer cell line, such as the cell line’s Achilles ID, and then processed the data to prepare it for analysis with the tools available on the SLKBase. We performed median-centering normalization of the gene expression data. Specifically, to normalize the expression value of a gene within a cell line, we subtracted the expression value by the median expression of the gene among all breast cancer cell lines. Along with expression data, we obtained copy number amplification, mutation data (COSMIC hit count), and CRISPR scores of gene dependency effects that were calculated using the CERES method^54^. We followed DepMap by considering hits in the CRISPR screens to be those having CERES scores of ≤−0.5.

### Development of MySQL database for breast cancer cell lines

We developed a MySQL database to store the functional genomics data for the breast cancer cell lines. This database is hosted on Google App Engine. The database contains tables for cell lines, genes, and proteins. Gene tables contain basic information from Entrez (gene name, symbol, ID) as well as a Boolean indicator if the gene is annotated as an oncogene by OncoKB. This allows us to unify gene essentiality information with a manual oncogene annotation. The database also contains linking tables between cell lines and genes that store the functional genomics data of a gene within a cell line (and likewise for proteomics data). For example, BCL2L1 has a linking table with each of the cell line tables; this linking table contains values for fold change, CNA, number of COSMIC mutations, etc.

### Development of R-Shiny apps for mining the database

We used the Shiny R package (https://shiny.rstudio.com) to develop the tools available on the SLKBase website and the tools are deployed on RStudio’s shinyapps.io (https://shinyapps.io). Each of the apps uses the R package “RMySQL” to query the MySQL database for functional genomics data. Furthermore, the Pathway Engine tools utilize the “Pathview” package to map the genomics data to genes within KEGG pathways.

The pathway essentialness algorithm was implemented in Python. Our implementation uses the NetworkX Python library to store the graph structure of KEGG pathways. We wrote scripts to parse the KEGG Markup Language (KGML) and convert them to the format of an adjacency matrix suitable for input to NetworkX.

### Development of the SUM breast cancer cell line Knowledge Base

The SLKBase web site was developed using WordPress tools and the HitMag style.

### Reporting summary

Further information on research design is available in the Nature Research Life Sciences Reporting Summary linked to this article.

## Supplementary information

Supplementary Material

Reporting Summary Checklist

## Data Availability

All the data sets supporting the findings of this study are publicly available in the SLKBase platform here: https://sumlineknowledgebase.com/. RPPA data, drug sensitivity data, Alpelisib response data, and data on dose response are also in the figshare repository, as part of this data record (10.6084/m9.figshare.12497630)^26^. The data from the 27 additional breast cancer cell lines that were incorporated into our database are available from DepMap portal (https://depmap.org/portal/), a publicly available data repository.

## Code availability

The R-shiny scripts that run the data mining tools on the web site are available at https://github.com/couchds/SLKBase-tools.
