# Supplementary material for: Development and implementation of the SUM breast cancer cell line functional genomics knowledge base
Source: NPJ Breast Cancer. 2020 Jul 21;6:30. doi: 10.1038/s41523-020-0173-z (PMC7374090; doi:10.1038/s41523-020-0173-z)
Supplement: Supplementary file 1 — Supplementary Material [file 41523_2020_173_MOESM1_ESM.pdf]

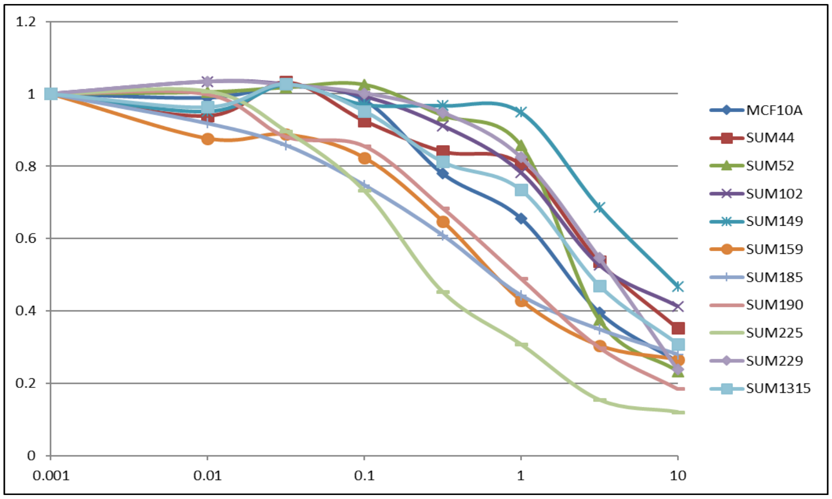

Supplementary figure 1. Response of the SUM cell line panel and MCF-10A cells to the Class-I alpha specific PI3'K inhibitor Alpelisib. Cells were seeded in 6 well plates and treated with half-log increasing concentrations of drug for 72 hours. The number of viable cells per well was determined in triplicate wells and plotted. These data were used to determine the IC50 for this drug for each cell line

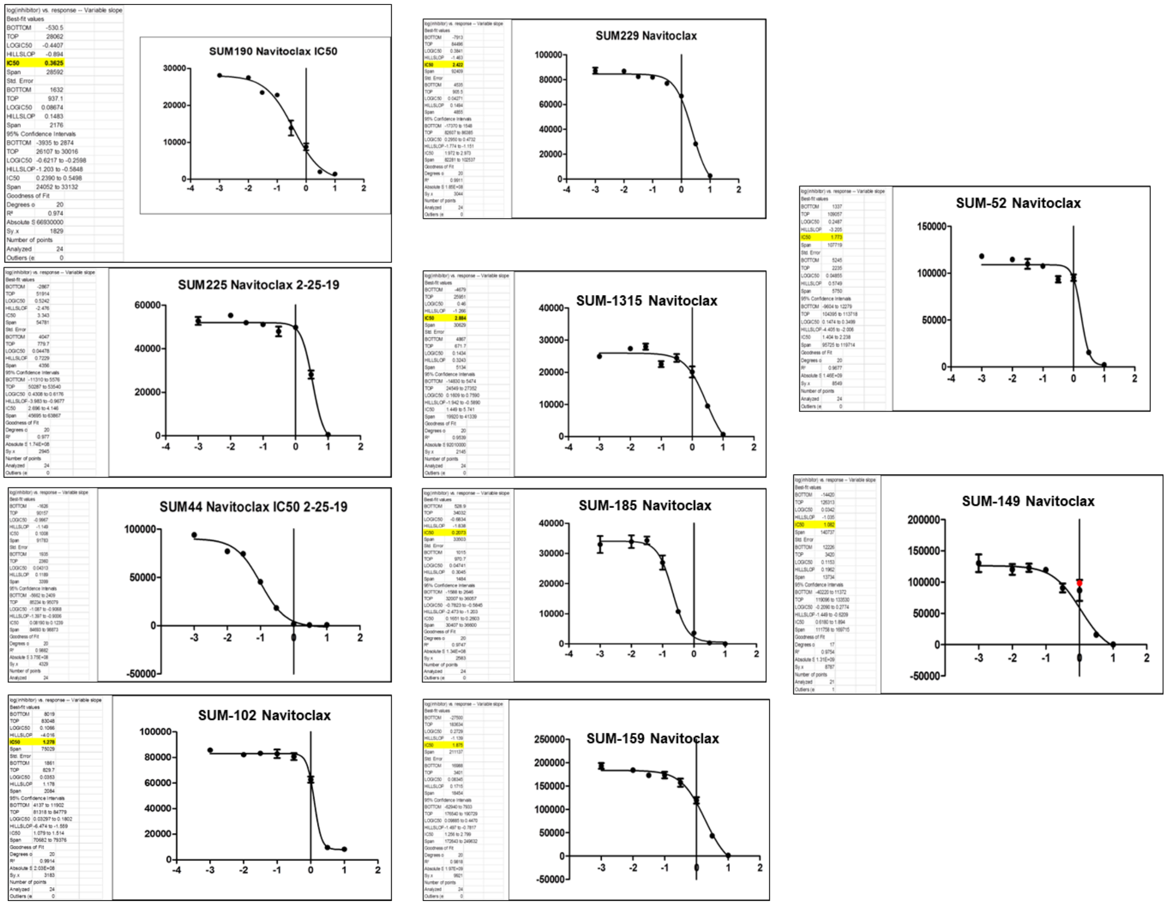

Supplementary figure 2. Individual concentration response curves for the SUM cell line panel to Navitoclax. Cells were plated in six well plates and treated for 72 hours with increasing concentrations of Navitoclax and the number of viable cells per well was determine using the Celligo Cell Analyzer

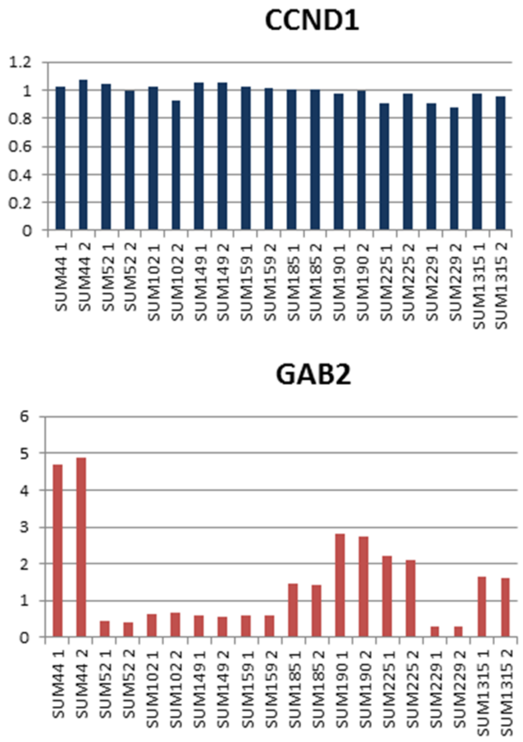

Supplementary Figure 3. Proteomic analysis of CCND1 and GAB2 across the cell line panel showing that for SUM-44 and SUM-190 cells, both of which exhibit the 11q14 amplicon, only GAB 2 is overexpressed at the protein level, suggesting that it is the driver from this

Supplementary Table 1. Overall Oncogene Signature for SUM-185 cells

GeneSymbol	QuantLog	QuantLogRank	ScreenHit	LogFoldChange	DnaAmp	Mutation	OccurencesInCosmic	ExistingDrugs
BCL2L1	5.8	5	1	1.16583842	1.1286		0	Obatoclax Mesylate, Navitoclax, TW 37
ID1	2.88	194	1	-0.69357046	1.1286		0	
ACSS1	2.88	195	1	1.24251594	1.0234		0	
ANKRD17	2.84	213	1	0.94384974	0.864		0	
NDUFA13	2.84	216	1	0.48131581	0.9679		0	
POFUT1	2.76	252	1	0.6381823	1.1286		0	
FGFR3	2.75	261	1	2.87313456	1.1797		0	PD173074
ISYNA1	2.47	542	1		0.9679		0	
PIK3CA	2.45	579	1			PIK3CAp.H1047R	1889	ZSTK474, PI-103, A66, BKM120
NKX2-2	2.3	876	1	1.55802764	1.0234		0	
GPT2	2.29	911.5	1		0.9666		0	
CTBP1	2.28	938	1		1.1797		0	
SSTR4	2.26	980	1	0.12581405	1.0234		0	
HSPBP1	2.26	997	1		0.9871		0	
COX4I2	2.21	1159	1	-0.07737755	1.1286		0	
IDUA	2.19	1245	1		1.1797		0	

Supplementary Table 2. Functional Oncogene Signature for SUM-185 cells

GeneSymbol	QuantLog	QuantLogRank	ScreenHit	LogFoldChange	DnaAmp*	Mutation	OccurencesInCosmic	ExistingDrugs
BCL2L1	5.8	5	1	1.165838	1.1286		0	Obatoclax Mesylate, Navitoclax, TW 37
FGFR3	2.75	261	1	2.873135	1.1797		0	PD173074
PIK3CA	2.45	579	1			PIK3CAp.H1047R	1889	ZSTK474, PI-103, A66, BKM120

Supplementary Table 3. Overall Oncogene Signature for SUM-190 cells

GeneSymbol	QuantLog	QuantLogRank	ScreenHit	LogFoldChange	DnaAmp	Mutation	OccurencesInCosmic	ExistingDrugs
PIK3CA	23.63	88	1			PIK3CAp.H1047R	1889	ZSTK474, PI-103, A66, BKM120
C8ORF59	19.25	125	1	0.05970123	0.8672			0
RPL30	18.93	131	1	0.59585438	0.8672			0
STK3	17.5	150	1	0.83040697	0.8672			0
RPL27	13.14	291	1	-0.37313864	3.0084			0
PLEKHF2	12.91	306	1	2.1968959	0.8672			0
NR1D1	11.56	370	1	1.6186231	3.4173			0
FNDC8	11.34	384	1		1.1823			0
TTC35	10.22	485	1	1.29444378	0.8672			0
SPAG1	9.72	534	1	1.35994785	0.8672			0
UCHL5IP	9.69	540	1		0.9519			0
UBN1	9.59	547	1	1.1575795	0.8509			0
LRP12	8.2	761	1	-0.79953172	0.8672			0
EPHA5	8.05	787	1	0	1.3009			0
RTN4RL2	7.47	931	1		1.3972			0
CXCL5	7.42	948	1	0.88665718	0.8759			0
MTCP1	7.41	951	1	0.28502328	1.2645			0
TMEM106A	7.36	965	1		3.0084			0

Supplementary Table 4. Functional Oncogene Signature for SUM-190 cells

GeneSymbol	QuantLog	QuantLogRank	ScreenHit	LogFoldChange	DnaAmp	Mutation	OccurencesInCosmic	ExistingDrugs
PIK3CA	23.63	88	1			PIK3CAp.H1047R	1889	ZSTK474, PI-103, A66, BKM120
EPHA5	8.05	787	1	0	1.3009		0	

Supplementary Table 5. Functional Oncogene Signature for SUM-225 [illegible]

GeneSymbol	QuantLog	QuantLogRank	ScreenHit	LogFoldChange	DnaAmp	Mutation	OccurencesInCosmic	ExistingDrugs
RAD21	11.57	441	1	0.8419769	0.9908		0	
ERBB2	6.68	1105	1	3.4614734	5.1635		0	Lapatinib, CP724714, CUDC-101, Afatinib
